# C/EBPβ Is a Transcriptional Regulator of Wee1 at the G_2_/M Phase of the Cell Cycle

**DOI:** 10.3390/cells8020145

**Published:** 2019-02-11

**Authors:** Ji Hae Lee, Jee Young Sung, Eun Kyung Choi, Hyun-Kyoung Yoon, Bo Ram Kang, Eun Kyung Hong, Byung-Kiu Park, Yong-Nyun Kim, Seung Bae Rho, Kyungsil Yoon

**Affiliations:** 1 Division of Translational Science, Research Institute, National Cancer Center, Goyang, Gyeonggido 411-769, Korea; jhlee5@ncc.re.kr (J.H.L.); museonthat@naver.com (E.K.C.); 74670@ncc.re.kr (H.-K.Y.); sirosama@hanmail.net (B.R.K.); ynk@ncc.re.kr (Y.-N.K.); sbrho@ncc.re.kr (S.B.R.); 2 Division of Clinical Research, Research Institute, National Cancer Center, Goyang, Gyeonggido 411-769, Korea; sungjy@ncc.re.kr (J.Y.S.); bkpark@ncc.re.kr (B.-K.P.); 3 Department of Pathology, National Cancer Center, Goyang, Gyeonggido 411-769, Korea; hongek@ncc.re.kr

**Keywords:** cell cycle, lung cancer, C/EBPβ, G_2_/M arrest, Wee1, Y15-pCDK1

## Abstract

The CCAAT/enhancer-binding protein β (C/EBPβ) is a transcription factor that regulates cellular proliferation, differentiation, apoptosis and tumorigenesis. Although the pro-oncogenic roles of C/EBPβ have been implicated in various human cancers, how it contributes to tumorigenesis or tumor progression has not been determined. Immunohistochemistry with human non-small cell lung cancer (NSCLC) tissues revealed that higher levels of C/EBPβ protein were expressed compared to normal lung tissues. Knockdown of C/EBPβ by siRNA reduced the proliferative capacity of NSCLC cells by delaying the G_2_/M transition in the cell cycle. In C/EBPβ-knockdown cells, a prolonged increase in phosphorylation of cyclin dependent kinase 1 at tyrosine 15 (Y15-pCDK1) was displayed with simultaneously increased Wee1 and decreased Cdc25B expression. Chromatin immunoprecipitation (ChIP) analysis showed that C/EBPβ bound to distal promoter regions of *WEE1* and repressed *WEE1* transcription through its interaction with histone deacetylase 2. Treatment of C/EBPβ-knockdown cells with a Wee1 inhibitor induced a decrease in Y15-pCDK1 and recovered cells from G_2_/M arrest. In the xenograft tumors, the depletion of C/EBPβ significantly reduced tumor growth. Taken together, these results indicate that Wee1 is a novel transcription target of C/EBPβ that is required for the G_2_/M phase of cell cycle progression, ultimately regulating proliferation of NSCLC cells.

## 1. Introduction

CCAAT/enhancer-binding protein β (C/EBPβ) is a member of the basic leucine zipper (bZIP) class of transcription factors and is involved in regulating cell growth, differentiation, inflammation, metabolism, survival, and tumorigenesis [1,2,3,4,5]. The growth-regulatory function of C/EBPβ has been reported to inhibit [6,7] or promote [8,9,10,11] cell proliferation, both in normal [6,8,9,10] and cancer cells [7,9,11]. For example, in C/EBPβ knock-out mice, hyperproliferation of epidermal keratinocytes was observed [6], but cell proliferation of B lymphocytes and gastric mucosa was decreased [8,9]. Thus, it is important to investigate how the function of C/EBPβ is determined in the cellular context and to determine what elicits these opposite growth responses including upstream regulators and downstream effectors.

C/EBPβ was shown to be required for carcinogen-induced mouse skin tumorigenesis by regulating p53-induced apoptosis upon carcinogen treatment [12,13], even though it induced differentiation of epidermal keratinocytes in normal physiology [6]. Reduced skin-tumorigenic potential was also observed in v-Ha-Ras transgenic mice when C/EBPβ was deficient, thereby suggesting C/EBPβ plays an oncogenic role in the downstream Ras signaling pathway [12]. The analysis of gene expression patterns of human cancers revealed that C/EBPβ is involved in cyclin D1-induced oncogenic signature [14]. Furthermore, increased expression of C/EBPβ and its oncogenic roles have been reported in breast, ovarian, colorectal, renal, and gastric cancers [15,16,17,18,19,20]. However, its detailed mechanism and function are yet to be determined.

The cell cycle is tightly controlled to prevent incorrect DNA replication or immature cell division that induces genomic instability, a hallmark of cancer [21]. Cell cycle progression is proceeded by cyclin-dependent kinases (CDKs) and their partner cyclins which regulate the activity of CDKs. Cyclin D-CDK4 and cyclin D-CDK6 play a role in G_1_ progression [22], and cyclin E-CDK2, cycle A-CDK2, and Cyclin B-CDK1 regulate the G_1_/S transition, S phase progression, and the G_2_/M transition, respectively [23,24,25]. CDK activity is also regulated by activating phosphorylation by CDK-activating kinase [26] and inhibitory phosphorylation by Wee1 and Myt1 [27]. Dephosphorylation of inhibitory phosphorylation by Cdc25 activates CDK activity and enables cells to continue cell cycle progression [27]. CDK inhibitors including the INK4 family that consists of p15, p16, p18, and p19 [28], and the Cip/Kip family comprising p21, p27 and p57 [29,30,31] also control CDK activity. In human cancers, mutation or dysregulated expression of genes involved in cell cycle regulation are frequently observed [21], which is attributed to accelerated cancer cell growth, leading to more malignant progression.

Lung cancer is the leading cause of cancer death worldwide and is also the most frequently diagnosed [32]. Non-small cell lung cancers (NSCLCs) account for approximately 85% of human lung cancer, and among them, lung adenocarcinoma and lung squamous cell carcinoma are most prevalent [33]. In NSCLC, altered regulation of cell cycle proteins including inactivation of p16, reduced expression of p27 and Rb, and overexpression of cyclin D were reported [21]. In addition, high expression of cyclin E and cyclin A, and low expression of Rb were correlated with unfavorable prognosis in NSCLC patients [34]. These observations imply that dysregulation of cell cycle is critical to lung cancer development, and altered cell cycle regulatory proteins could be important therapeutic targets or prognostic markers. 

Lung cancer is a genetically heterogeneous disease, and in lung adenocarcinoma, K-RAS and epidermal growth factor receptor (EGFR) are most commonly activated by mutations [33]. Both oncogene products potentiate cell cycle progression upon mitogenic signaling, resulting in more aggressive phenotypes. As C/EBPβ has been reported to mediate several oncogenic signaling pathways, including receptor tyrosine kinases or activated Ras [12,35], and the growth-regulatory function of C/EBPβ in lung cancer has not been fully defined, we investigated the role of C/EBPβ in human NSCLCs. 

In this paper, we report that C/EBPβ is frequently overexpressed in lung cancer tissues compared with normal lungs tissues, and regulates cell proliferation by mediating cell cycle progression at the G_2_/M phase in NSCLC cells.

## 2. Materials and Methods

### 2.1. Cell Culture and Reagents

A549, Calu-6, NCI-H1299, NCI-H1703, NCI-H1975, NCI-H23, NCI-H460, HCC2279, NCI-H522, A427, Calu-3, NCI-H358, HCC827 and BEAS2B were obtained from the American Type Culture Collection (Rockville, MD, USA). HCC95 and HCC1588 were obtained from the Korean Cell Line Bank (Seoul, Korea), and Normal human bronchial epithelial (NHBE) purchased from Lonza. A549, Calu-6, NCI-H1299, NCI-H1703, NCI-H1975, NCI-H23, NCI-H460, HCC2279, NCI-H522, NCI-H358, HCC827, HCC95, HCC1588, and BEAS2B were cultured with RPMI1640. Calu-3 and A427 were cultured with Dulbecco’s modified eagle medium (DMEM). NHBE was cultured with bronchial epithelial cell growth medium (BEGM) BulletKit (Lonza, Walkersville, MD, USA). All cell lines were maintained in media supplemented with 10% fetal bovine serum (FBS) and 1× penicillin-streptomycin, and cultured under standard conditions at 37 °C in a humidified atmosphere of 95% air and 5% CO_2_. Stock solutions of the Wee1 inhibitor MK-1775 (Selleck Chemicals, Houston, TX, USA) were dissolved in dimethyl sulfoxide (DMSO) and added to the media at the indicated concentrations (100 nM). Doxycycline (D9891) was purchased from Sigma-Aldrich (St. Louis, MO, USA). Matrigel was purchased from Corning Inc (Corning, NY, USA). 

### 2.2. siRNA Transfection and Generation of Conditional Knockdown Cell Lines

Cells were seeded in 6-well culture plates at a density of 2 × 10^5^ cells per well, and grown for 16 h before transfection with 20 nM of small interfering RNA (siRNA) for 48 h. The sequences of the CCAAT/enhancer binding protein β (C/EBPβ) siRNA are as follows: siC/EBPβ #1, 5′-CCTCGCAGGTCAAGAGCAA-3′; siC/EBPβ #2, 5′-CCAAGAAGACCGTGGACAA-3′. SiRNA duplexes were transfected using Lipofectamine 2000 (Invitrogen, Waltham, MA, USA) following the manufacturer’s protocol. To generate doxycycline-inducible C/EBPβ-knockdown cell lines, the C/EBPβ target sequences were cloned into the Tet-pLKO-Puro plasmid (Addgene plasmid #21915,). The Tet-on-shC/EBPβ target sequence is 5′-CACCCTGCGGAACTTGTTCAA-3′. To induce C/EBPβ-knockdown, doxycycline (100 ng/mL) was added.

### 2.3. Cell Proliferation Assay

Cells were plated in triplicate at 10% confluence in 24-well culture plates and transfected with negative control siRNA (siNC) or C/EBPβ siRNA (siC/EBPβ) at a final concentration of 20 nM. Cells were harvested and counted using a Coulter counter (Beckman Coulter, Brea, CA, USA) in 24 h intervals. An IncuCyte live-cell imaging system (Essen Bioscience, Ann Arbor, MI, USA) was used to measure the proliferation of Tet-on-shC/EBPβ or shNC cells using the cell confluence approach.

### 2.4. Live/Dead Cell Staining

Cells were stained using LIVE/DEAD™ Viability/Cytotoxicity Kit (Thermo Scientific, Rockford, IL, USA) following the manufacture’s protocol. Images were obtained by an Operetta High Content Screening (HCS) System (PerkinElmer, Waltham, MA, USA) and analysis was performed using Harmony 3.5.2 software (PerkinElmer).

### 2.5. Cell Cycle Analysis

For thymidine double block [36], cells were seeded at 2 × 10^5^ cells per well in 6-well culture plates and treated with thymidine at 2 mM for 14 h. Cells were then washed, supplemented with normal media for 12 h, and treated with 2 mM thymidine for another 14 h. The cells were harvested at 2 h intervals up to 16 h and 26 h after release. Afterward, cells were harvested and fixed in 75% (*v*/*v*) cold ethanol at −20 °C for at least 2 h. The fixed cells were collected by centrifugation and resuspended in propidium iodide (PI) Staining Buffer (Sigma, St. Louis, MO, USA) to stain DNA and analyzed for DNA content on a flow cytometry (FACSCaliber; Becton Dickinson, Franklin Lakes, NJ, USA).

### 2.6. Live Cell Fluorescence Imaging for Analysis of Motisis

A549 cells were seeded in 12-well culture plates and transfected with negative control siNC or siC/EBPβ. After 24 h, cells were stained with NucBlue^®^ live reagent (Hoechst 33342 dye, Thermo Scientific, Rockford, IL, USA) for 20 min following matufcturer’s protocol, then replaced with fresh culture medium. Images were taken by Operetta High Content Screening (HCS) System (PerkinElmer, Waltham, MA, USA) every 10 min for 48 h under 200× magnification.

### 2.7. Western Blot Analysis

Whole-cell lysates were prepared in radioimmunoprecipitation assay (RIPA) buffer, supplemented with protease inhibitor cocktail, phosphatase inhibitor (Calbiochem, San Diego, CA, USA), phenylmethylsulfonyl fluoride (PMSF), and dithiothreitol (DTT). Protein concentrations were determined using a micro bicinchoninic acid (BCA) protein assay kit (Thermo Scientific, Rockford, IL, USA). Equal amounts of protein were separated by 6–12% Sodium dodecyl sulphate-polyacrylamide gel electrophoresis (SDS-PAGE) and transferred to a polyvinylidene difluoride (PVDF) membrane (BioRad, Hercules, CA, USA) using a wet transfer device (BioRad, Hercules, CA, USA). The following antibodies were used in this study: C/EBPβ (sc-7962), MAD2 (sc-6329), and β-actin (sc-477778) (all obtained from Santacruz Biotechnology, Santa Cruz, CA, USA); anti-Cdc2/Cdk1 (06-923), phospho-Cdk1 (Tyr15) (#9111), Cdc25B (#9525), Wee1 (#4936), and Cdc25A (#3652) (all obtained from Cell Signaling Technology Beverly, MA, USA); and anti-CyclinB1 (05-373) and Cdc25C (05-507) (all obtained from Millipore, Bedford, MA, USA).

### 2.8. Quantitative Real-Time PCR

Total RNA was prepared by using Trizol (Ambion, Life Technologies, Carlsbad, CA, USA) and cDNA was synthesized using Moloney murine leukemia virus (M-MLV) reverse transcriptase (Invitrogen, Waltham, MA, USA). Real-time polymerase chain reaction (PCR) was performed using LightCycler^®^ 96 Real-Time PCR System (Roche, Basel, Switzerland). Each reaction was performed with 10 ng cDNA by using SYBR Green. Primer sequences used for PCR were as follows: Wee1, (F: 5′-TTCAATGAGGAGACTTGCCTG-3′ and R: 5′-ACAACAACAATCTGAGGTGCC-3′); Cdc25B, (F: 5′-GTGAGGAAGTTTCAGAACAGTCCG-3′ and R: 5′-TGGGAGGCTTGTCGCATTTG-3′), and GAPDH, (F: 5′-TGATGACATCAAGGTGGTGAAG-3′ and R: 5′-TCCTTGGAGGCCATGTGGGCCAT-3′). PCR reactions were performed as follows: 95 °C denaturation for 5 min, followed by 40 cycles at 94 °C for 10 s, 60 °C for 10 s, 72 °C for 10 s, followed by a 9-min extension at 72 °C. For displaying PCR products on agarose gel, PCR cycle was reduced to 30 cycles.

### 2.9. Chromatin Immunoprecipitation

The chromatin immunoprecipitation (ChIP) assay was performed using the EZ-ChIP assay (Upstate Biotechnology, Lake Placid, NY, USA) following the manufacturer’s protocol. Briefly, formaldehyde was added at a final concentration of 1% directly to cell culture media. Fixation proceeded at RT for 10 min and was stopped by the addition of glycine to a final concentration of 0.125 M. The cells were collected by centrifugation and rinsed in cold phosphate-buffered saline (PBS). The cell pellets were resuspended in hypotonic buffer containing 0.5 mM PMSF, protease inhibitor cocktail, and incubated on ice for 15 min. The nuclei were collected by micro-centrifugation and then resuspended in SDS lysis buffer (1% SDS, 10 mM EDTA, 50 mM Tris-HCl (pH 8.1), 0.5 mM PMSF, and protease inhibitor cocktail). The samples were sonicated to an average length of 300–500 bp with a S220 Focused-ultrasonicator (Covaris, Woburn, MA, USA). Chromatin immunoprecipitation was performed with anti-C/EBPβ (sc-150X, Santacruz Biotechnology, Santa Cruz, CA, USA) or HDAC2 (sc-9959X, Santacruz Biotechnology, Santa Cruz, CA, USA) and protein G agarose. ChIP products were eluted and DNA was recovered from reverse crosslinking and purification. C/EBPβ binding to specific sites on the *Wee1* promoter was analyzed by quantitative real-time PCR (qRT-PCR). Primers for PCR analysis were follows: R1 (F, 5′-CAGTCTAGTTGTGGAGAGGCA-3′ and R, 5′-CCTGCCACTCCTGATGACAAA-3′); R2 (F, 5′-CAGTGTGTGCTTTACTCAGAGGAG-3′ and R, 5′-CTCCAGCAACCAGCACTGT-3′); R3 (F, 5′-TCAAAGTGCAAGGCTCATGT-3′ and R, 5′-TTTGCAGAATCCACATGCTT-3′); R4 (F, 5′-TGCTGATGAACATGCGGTGA-3′ and R, 5′-CTGCCTATTGGCCTCAGGAA-3′); *GAPDH* exon (F, 5′-TCTATAAATTGAGCCCGCAGC-3′ and R, 5′-GCGACGCAAAAGAAGATGC-3′).

### 2.10. Luciferase Reporter Assay

The constructs of *Wee1* promoter region were cloned into pGL3-promoter firefly luciferase vector (Promega, Madison, WI, USA), which were named R3 (−4932 to −4679), and R2 (−4543 to −4380), C/EBPβ and HDAC2 cDNA clones purchased from OriGene were sub-cloned into pcDNA3.1 vector (Invitrogen, Waltham, MA, USA). Cells were seeded in 24-well plates and co-transfected with reporter vectors and pcDNA3.1 or pcDNA3.1-C/EBPβ and/or pcDNA3.1-HDAC2 as indicated using Lipofectamine 2000 (Invitrogen). After 48 h, cells were harvested. Firefly luciferase activities were determined using the Luciferase assay system kit (Promega, Madison, WI, USA), as described by the manufacturer, with a luminescence plate reader (VICTOR™ X, PerkinElmer, Waltham, MA, USA). The firefly luciferase activity was normalized for transfection efficiency with protein measurement using a BCA protein assay. Data are expressed as relative luciferase activity/μg protein.

### 2.11. Immunoprecipitation 

Cells were lysed in cell lysis buffer (20 mM Tris–HCl pH8.0, 150 mM NaCl, 1 mM Na_2_EDTA, 1 mM EGTA, 1% Triton, 2.5 mM sodium pyrophosphate, and 1 mM β-glycerophosphate). Each cell lysate (1 mg) was incubated with C/EBPβ monoclonal antibody (Santacruz Biotechnology, Santa Cruz, CA, USA) overnight at 4 °C. Following incubation, protein was immunoprecipitated using protein G agarose beads (GE Healthcare, Chicago, IL, USA) for 2 h at 4 °C with gentle rotation. The immunoprecipitates were washed three times with lysis buffer and boiled in 20 μL of 1× SDS sample buffer for 5 min at 95 °C. After centrifugation, the supernatant was analyzed using Western blot.

### 2.12. Xenograft Mouse Model and siRNA Delivery

A549 (5 × 10^6^) cells were suspended in 100 μL PBS and mixed with 50 μL Matrigel (Corning Inc.). The mixtures were implanted subcutaneously into 6-week-old athymic nude mice. When the tumor size reached 60 to 80 mm^3^, the dilute siRNA solution in sterile PBS (50 μL) was directly injected into the xenograft tumor via electroporation using NEPA21 Super Electroporator (Nepa gene Co., Chiba, Japan). The tumor size was monitored every 7 days up to 7 weeks. Tumor diameters were measured twice a week and the volume was calculated with the following formula: V (mm^3^) = longest diameter × shortest diameter ^2^/2.

### 2.13. Immunohistochemical Staining for Xenograft Tumor

Xenograft tumors were removed and fixed in 10% formalin, embedded in paraffin, and cut into 4-μm sections. The sections were used for immunohistochemical staining performed with the automated instrument Discovery XT (Ventana Medical Systems, Inc., Tucson, AZ, USA) using anti-C/EBβ (sc-150, Santacruz Biotechnology, Santa Cruz, CA, USA), anti-Wee1 (ab37597), Cdc25B (ab70927), phospho-Cdk1(Tyr15) (ab133463), anti-Ki67 (ab15580) (all from Abcam, Cambridge, UK), and cleaved caspase3 (#9661, Cell signaling Technology Beverly, MA, USA).

### 2.14. Immunohistochemical Staining for Lung Cancer Tissue Microarray

Lung tissue arrays [CCN5, Human, Normal lung (59 adjacent normal lung tissues matching CC5, 1 carbon); CC5, Human, Lung cancer (59 NSCLC tissues, 1 carbon); CCA4 Human, Lung cancer-metastasis-normal (36 NSCLCs, 1 missing NSCLC, 2 small cell lung cancers (SCLCs), 1 malignant mesothelioma; 9 metastatic tissues matching 9 among 36 NSCLCs, 1 metastatic tissue matching 1 among 2 SCLCs; 9 normal lung tissues matching 9 among 36 NSCLC, 1 carbon] were purchased from Superbiochips Laboratories (Seoul, Korea) [37]. Total number of tissues on 3 microarrays was 68 for adjacent normal lung tissues, 95 for NSCLC tissues and 9 for metastatic tissues from 95 patients. Each array contained 59 sections of 4 μm thickness obtained by surgical resection and one carbon for orientation. The sections were used for immunohistochemical staining performed with the Ventana BenchMark XT Staining systems (Ventana Medical Systems, Inc.) using C/EBPβ antibody (1:30, sc-150 Santacruz Biotechnology, Santa Cruz, CA, USA) and the UltraView Universal DAB detection kit (Ventana Medical Systems, Inc.). Parallel sections incubated with normal IgG antibody instead of primary antibodies were used for negative controls. The stainings were scored from 0 to 4 based on the intensity and proportion of positive staining in a tissue field. Stained tissue arrays were reviewed by two experienced medical pathologists. To obtain representative images, slides were scanned by the Aperio ScanScope scanner (Aperio Technologies, Vista, CA, USA) and images were captured using Aperio ImageScope software.

### 2.15. Statistical Analysis

All data points represent average values and standard deviation (SD) or standard error (SE) obtained from three independent experiments performed in triplicate. Comparison between two groups was performed using Student’s *t*-test. The relationship between C/EBPβ expression and clinicopathologic characteristics was analyzed using the Student’s *t*-test. Statistical significance was defined as a *p* value < 0.05. For survival analysis, Kaplan–Meier Plotter [38] was used to investigate the association of C/EBPβ mRNA expression in overall survival and post-progression survival in lung cancer patients.

## 3. Results

### 3.1. Levels of C/EBPβ Protein in Human Lung Cancer Tissues Were Elevated

**Figure 1:**
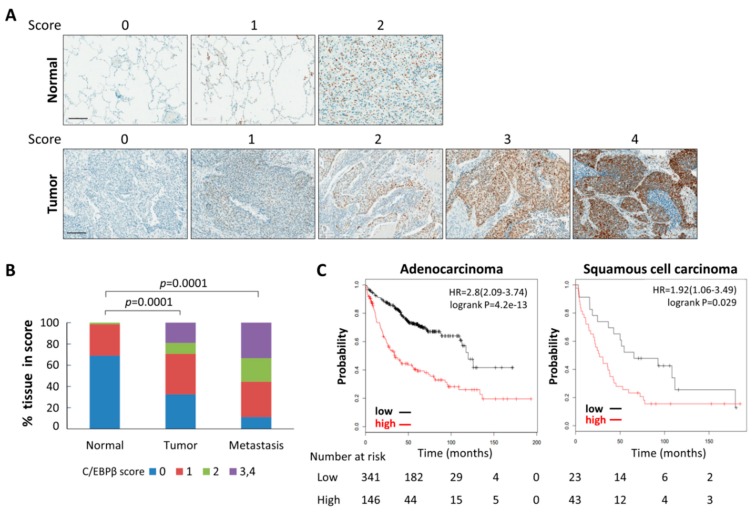
CCAAT/enhancer-binding protein β (C/EBPβ) expression in human lung cancer tissues. (**A**) Patients-derived lung cancer tissue arrays were examined for C/EBPβ expression using the immunoperoxidase method. Staining results were graded according to the intensity and proportion of positive area. Images were captured at a magnification of 200X by using the Aperio ImageScope software. Scale bars: 200 μm. (**B**) The histogram represents the percentage of the immunohistochemistry (IHC) score for C/EBPβ in 68 normal tissues, 95 primary, and 9 metastatic tumor tissues. The statistical significance was determined using the *t*-test, *p* < 0.05. (**C**) The association between C/EBPβ mRNA expression and overall survival of adenocarcinoma (whole dataset) and squamous cell carcinoma patients (GSE37745) was analyzed using the Kaplan–Meier Plotter. Hazard ratio (HR) significance was found with log-rank tests.

To explore the clinical significance of C/EBPβ in lung cancer, we examined the expression of C/EBPβ in patient-derived lung cancer tissue microarrays. Lung cancer tissues and adjacent normal lung tissue obtained from 95 patients were immunohistochemically stained for C/EBPβ. Representative pictures of C/EBPβ staining results with varying intensities (0 to 4) are shown in Figure 1A. As shown in Figure 1B and Table 1, the proportion of normal lung tissues with positive C/EBPβ staining was only 30.9%, whereas positive C/EBPβ staining increased up to 67.3% in the primary lung cancer tissues, including 29.4% of strong staining intensity (score 2–4). Scoring of C/EBPβ staining in each NSCLC subtype is listed in Figure S1A. Tumor samples from metastatic patients tended to display strong staining for C/EBPβ (score 2–3, 55.5%), and positive C/EBPβ staining of metastatic tissues was significantly higher than that of normal lung tissues (Figure 1B). Immunohistochemical analyses of primary NSCLC tissues showed that C/EBPβ expression was already enhanced from the early stages of lung cancer but there were no significant differences among different clinical stages, tumor, node, metastasis (TNM) stage, age, or sex (Table 2). A significant increase in C/EBPβ positive staining was observed in squamous cell carcinoma and other types of NSCLCs compared with lung adenocarcinoma. Using a public database, we also analyzed gene alteration status of *C/EBPB* in lung adenocarcinoma and lung squamous cell carcinoma, the most common subtypes of non-small cell lung cancers (NSCLCs). The *C/EBPB* gene was amplified by 1.22% and 1.57% and deleted by 0.1% and 0.17% in lung adenocarcinoma and lung squamous cell carcinoma, respectively, indicating that alteration of the C/*EBPB* gene is not frequent (Figure S1B). An association between clinical outcomes of lung cancer patients and C/EBPβ expression was examined using the Kaplan–Meier Plotter [38]. This revealed that the levels of C/EBPβ mRNA were inversely correlated with the overall survival of patients with lung cancers, adenocarcinomas, and squamous cell carcinomas (Figure 1C and Figure S2A). Additionally, increased expression of C/EBPβ mRNA was associated with poor post-progression survival in all lung cancer and adenocarcinoma patients (Figure S2A,B). All these data indicate that the expression of C/EBPβ is upregulated at the protein levels, which is a functional moiety, in human lung cancer and possibly correlated with clinical outcome of patients. 

### 3.2. Knockdown of C/EBPβ Inhibits Cell Proliferation Rates of NSCLC Cells

**Figure 2:**
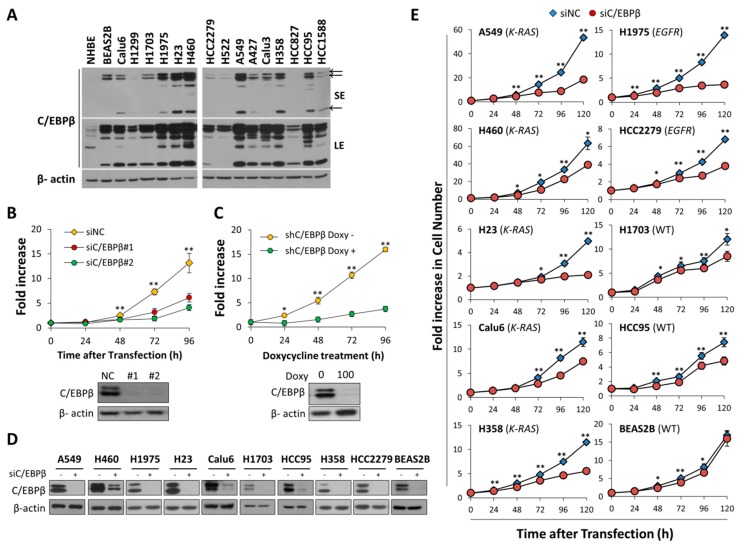
C/EBPβ promotes cell proliferation in various subtypes of non-small cell lung cancer (NSCLC) cell lines. (**A**) C/EBPβ protein levels in normal human bronchial epithelial cells (NHBE), immortalized human bronchial epithelial cells, BEAS-2B and various NSCLC cell lines were determined by Western blot analysis. Aenocarcinoma, A549, NCI-H1975, NCI-H23, NCI-H1703, NCI-H522, A427, Calu-3, NCI-H358; adeno-squamous cell carcinoma, HCC2279; squamous cell carcinoma, HCC95, HCC1588; large cell carcinoma, H460, NCI-H1299; anaplastic carcinoma, Calu-6. (**B**) Live cells of A549 transfected with si-Negative Control (siNC), siC/EBPβ #1, or siC/EBPβ #2 were counted with trypan blue staining at indicated times after transfection. Data are presented as fold increase. (**C**) Doxycycline-inducible shC/EBPβ cells using A549 were generated and treated with or without doxycycline (100 ng/mL). Using the IncuCyte live cell imaging system, proliferation cells was monitored and quantified by the percentage of cell confluence. (**D**) The protein levels of C/EBPβ were detected by Western blotting to check C/EBPβ-knockdown in each cell line. (**E**) Cell number of lung cancer cell lines transfected with siNC or siC/EBPβ (#1 + #2) was counted using a Coulter counter at intervals of 24 h up to 120 h after siRNA transfection. *K-RAS*: mutant *K-RAS*/wild-type *EGFR*, *EGFR*: wild-type *K-RAS*/mutant *EGFR*, WT: wild-type *K-RAS*/wild-type *EGFR*. Data are presented as mean ± standard deviation (SD). The statistical significance was determined using *t*-tests, * *p* < 0.05, ** *p* < 0.01.

In this research, we measured the levels of C/EBPβ protein in normal human bronchiolar epithelial cells (NHBE), immortalized human bronchial epithelial cells (BEAS-2B), and various subtypes of NSCLC cell lines. Different subtypes of lung cancers are known to be distinct in origin and in disease progression [39]. NHBE cells expressed C/EBPβ protein in marginal amounts compared with BEAS-2B cells and NSCLC cell lines tested (Figure 2A). There was no significant association between C/EBPβ protein levels and specific NSCLC subtype.

K-Ras and EGFR is frequently activated by mutation and they are mutually exclusive in lung adenocarcinomas [33], and these oncogenes might act as upstream regulator of C/EBPβ. However, the levels of C/EBPβ protein were not very different among adenocarcinomas with mutant *K-RAS* (A549, H23, A427, NCI-H358), mutant *EGFR* (NCI-H1975, HCC827) and wild-type *K-RAS* and *EGFR* (NCI-H1703, NCI-H522, Calu-3) [40,41,42,43,44], even though A549 and H23 cells displayed relatively high levels of C/EBPβ (Figure 2A). 

To examine the function of C/EBPβ in lung cancer cells, we knocked down C/EBPβ using two different siRNAs in NSCLC cell lines. Transfection of each siC/EBPβ dramatically suppressed proliferation of A549 cells with C/EBPβ down-regulation (Figure 2B). We also employed doxycycline-inducible shRNA targeting another sequence of *C/EBPβ* and observed that doxycycline treatment decreased C/EBPβ protein and cell proliferation (Figure 2C). 

To exclude the possibility that retarded cell proliferation induced by C/EBPβ–knockdown was limited to A549 cells, we knocked down C/EBPβ in several NSCLC cell lines with decent levels of C/EBPβ protein (Figure 2D). Consistent with results shown in Figure 2B,C, cell proliferation rates were significantly slower in siC/EBPβ-transfected NSCLC cells than in negative control-siRNA (siNC)-transfected cells (Figure 2E). Neither histological subtypes nor *EGFR* and *K-Ras* mutation status of the cell lines produced significant differences in the growth arrest induced by C/EBPβ-knockdown (Figure 2E). NSCLC subtypes and mutational status of *K-RAS* and *EGFR* in each cell line are listed in Table S1. Although C/EBPβ-knockdown attenuated cell proliferation in BEAS-2B cells, the degree of inhibition appeared minimal compared with NSCLC cell lines. These observations indicate that C/EBPβ is important for cell proliferation in NSCLC cells.

### 3.3. C/EBPβ-Knockdown Delayed G_2_/M Phase of the Cell Cycle Progression with Elevated Levels of Inhibitory Phosphorylation of CDK1

**Figure 3:**
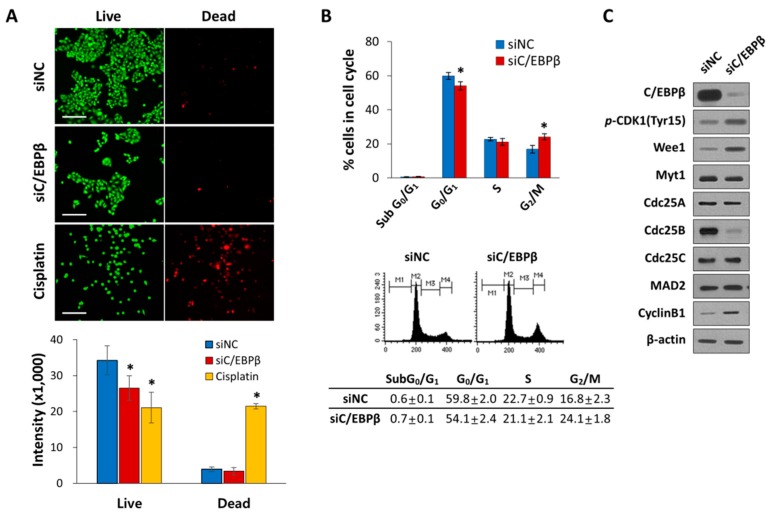
C/EBPβ-knockdown inhibits cell cycle progression. (**A**) A549 cells were transfectected with siNC or siC/EBPβ and incubated for 48 h. As a positive control for cell death, cells were treated with 20 M cisplatin for 48 h. Live cells were stained with green calcein-AM, while dead cells were stained with red ethidium homodimer-1 (EthD-1). Cell images were taken at a magnification of 100X using an Operetta High Content Screening (HCS) System. Scale bar: 200 μm. (**B**) A549 cells were transfected with control siRNA or C/EBPβ siRNA for 48 h. The cell cycle was analyzed by fluorescence-activated cell sorting (FACS) after DNA staining with propidium iodide (PI). M1: subG_0_/G_1_, M2: G_0_/G_1_, M3: S, and M4: G_2_/M phase. Percentage of cells in each cell cycle phase is shown as a bar graph. (**C**) Whole cell lysates were prepared 48 h after transfection and the levels of the G_2_/M cell cycle-related proteins in control or C/EBPβ-knockdown cells were analyzed by Western blotting. β-actin was used as a loading control. Data are presented as mean ± SD. Statistical significance was determined using the *t*-test, * *p* < 0.01.

Next, we investigated whether inhibition of cell proliferation was due to increased cell death or inhibited cell cycle progression in the C/EBPβ-knockdown cells. There was little difference in cell death between control and C/EBPβ-knockdown cells (Figure 3A), indicating cell death was not involved in C/EBPβ-knockdown-induced inhibition of cell proliferation. We then examined the role of C/EBPβ in cell cycle regulation. We analyzed the cell cycle of C/EBPβ-knockdown cells by flow cytometry, which showed a significant decrease in the population of cells in the G_0_/G_1_ and a simultaneous increase in the G_2_/M phase of the cell cycle compared with the control (Figure 3B). G_2_/M progression of the cell cycle is mainly regulated by cyclin B/CDK1 [45]. Activity of CDK1 is regulated by its phophorylation. The Wee1 and Myt1 kinases phosphorylate CDK1 at tyrosine 15 (Tyr15), thereby inhibiting CDK1. Dephosphorylation of Tyr15 by cell division cycle 25 (Cdc25) phosphatase is required for the activation of CDK1 and further entry into mitosis [46,47,48,49,50]. Knockdown of C/EBPβ increased phosphorylation of CDK1 at the tyrosine 15 residue (Y15-pCDK1) with a simultaneous increase in the levels of Wee1 and cyclin B1 and a decrease in the levels of Cdc25B (Figure 3C). The expression of Cdc25A and Cdc25C remained unchanged between the groups. C/EBPβ-knockdown did not appeared to affect the protein levels of Myt1 and mitotic arrest deficient 2 (Mad2), mitotic spindle checkpoint component [51]. Taken together, these data indicate that C/EBPβ-knockdown cells undergo a delay in the G_2_/M phase of the cell cycle, displaying elevated Y15-pCDK1 possibly due to increased Wee1 and decreased Cdc25B.

**Figure 4:**
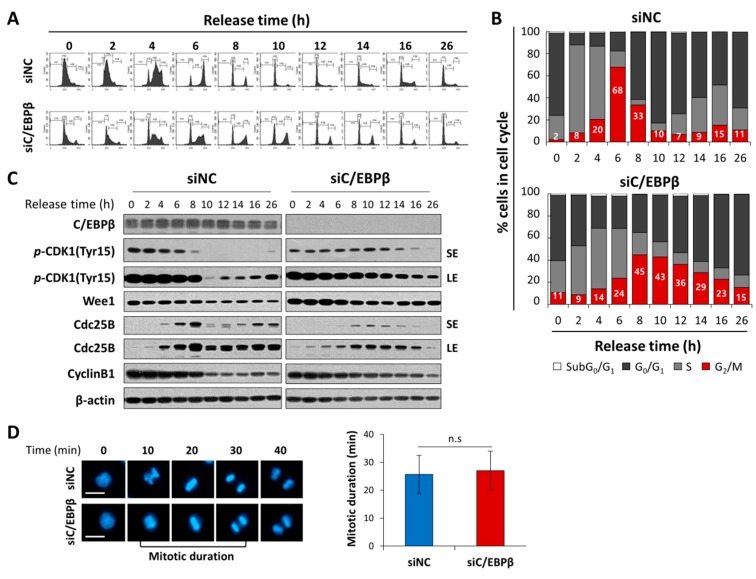
C/EBPβ knockdown delays G_2_/M-cell cycle transition. (**A**) A time course study of the cell cycle analysis was performed in control or C/EBPβ-knockdown A549 cells. Cells were released from thymidine double block-induced G_1_/S synchronization; and 0, 2, 4, 6, 8, 10, 12, 14, 16, and 26 h after releasing, cells were collected and stained with PI to measure DNA content using FACS. (**B**) The data are expressed as the percentage of cells in the subG_0_/G_1_, G_0_/G_1_, S, and G_2_/M phase at the indicated time points. (**C**) Whole cell lysates were prepared at the indicated times and Western blot analysis was performed for expression of proteins associated with the G_2_/M transition (Y15-pCDK1, Wee1, Cdc25B and Cyclin B1). SE; short exposure, LE; long exposure. (**D**) Mitotic duration of control or C/EBPβ-knockdown A549 cells stained with NucBlue^®^ was monitored by an Operetta High Content Screening (HCS) System. Representative images of siNC and siC/EBPβ-transfected cells from time lapse series were shown. Images were acquired every 10 min. Data are presented as mean ± SD of mitotic duration in 30 cells in each group. Mitotic duration was measured from nuclear envelope breakdown (prometaphase) to anaphase onsets [52]. Statistical significance was determined using the *t*-test; n.s., not significant. Cell images were taken at a magnification of 200X using an Operetta High Content Screening (HCS) System. Scale bar: 20 μm.

In order to observe a temporal progression in the G_2_/M phase of the cell cycle, we synchronized cells at the G_1_/S boundary using thymidine double block methods [36], and released cells into the cell cycle by removing thymidine. Control cells progressed into the cell cycle and accumulated in the G_2_/M phase 6 h after release at the highest levels, and subsequently moved into the G_1_ phase in just 2–4 h (Figure 4A,B). In contrast, C/EBPβ-knockdown cells reached the G_2_/M peak 8 h after release into the cell cycle, and stayed in the G_2_/M phase for much longer up to 16 h. Cell populations in the G_2_/M phase in an asynchronized condition (26 h) were significantly higher in C/EBPβ-knockdown cells compared with controls, consistent with results shown in Figure 3B. To determine the effect of C/EBPβ-knockdown on mitotic progression, we analyzed mitotic duration using time-lapse live cell imaging with nuclear Hoechst 33342 staining. As shown in Figure 4D, mitotic duration from nuclear envelope breakdown to anaphse onset [52] was not significantly different between control and C/EBPβ-knockdown cells, suggesting that a delay in the G_2_/M phase of the cell cycle progression displayed in C/EBPβ-knockdown cells was not due to a delay in the mitotic phase, but was rather due to prolonged stay in the G_2_ phase of the cell cycle. To confirm a delay in the G_2_-M transition of C/EBPβ-knockdown cells, we analyzed mitotic cell populations using flow cytometry along with phospho-histone H3 (Ser10) and PI staining. Phosphorylation of histone H3 (Ser10) is essential for mitotic condensation and thus considered a mitotic marker [53]. As shown in Figure S3, more C/EBPβ-knockdown cells were accumulated in the G_2_/M phase than control cells, consistent with results in Figure 3B, but mitotic cells with positive phospho-histone H3 (Ser10) staining decreased significatly in C/EBPβ-knockdown cells. Combined with the live cell imaging results, C/EBPβ seem to be important in the G_2_/M transition of the cell cycle. 

In the control cells, levels of Y15-pCDK1 started to decrease within 6 h and suddenly dropped 10 h after release when the most cells returned to the G_1_ phase (Figure 4C). A timely and transient increase in Cdc25B protein levels was correlated with a decrease in Y15-pCDK1, which is an important event for mitotic entry. However, Y15-pCDK1 stayed at higher levels for a longer time with an increase in Wee1 and a delayed and attenuated Cdc25B induction in the C/EBPβ-knockdown cells compared with control cells (Figure 4C). Taken together, our data demonstrate that C/EBPβ is an important mediator at the G_2_/M phase of the cell cycle progression by regulating the expression of Wee1 and Cdc25B, which critically modulates Y15-pCDK1.

### 3.4. C/EBPβ Regulates Wee1 Expression at the Transcription Levels

**Figure 5:**
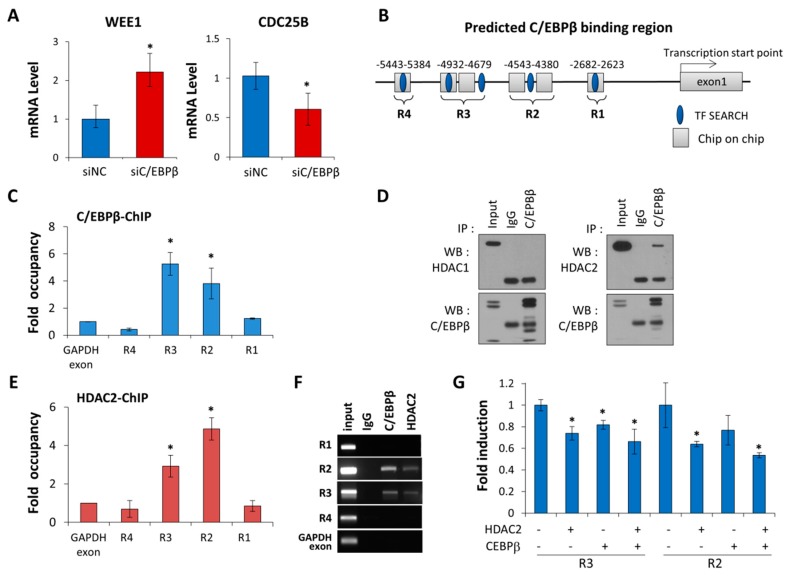
C/EBPβ regulates Wee1 expression at the transcription levels and interacts with HDAC2. (**A**) Quantitative real-time RT-PCR (qRT-PCR) was used to determine Wee1 and Cdc25B mRNA levels relative to the control gene *GAPDH* in C/EBPβ-knockdown A549 cells. Data are presented as mean ± SD. (**B**) The position of the four predicted C/EBPβ binding sites in the *WEE1* promoter are represented. C/EBPβ–ChIP on chip data and TFSEARCH based binding sites are indicated as a rectangle and an oval, respectively. The prediction of *WEE1* promoter regions was based on NCBI accession number (NC_000011.10, GRCh37.p11) (**C**) The C/EBPβ-ChIP assay followed by qRT-PCR on putative C/EBPβ binding regions on the *WEE1* promoter was performed to determine endogenous C/EBPβ occupancy at the specified region. The fold enrichment of C/EBPβ occupancy over *GAPDH* exon (negative control) is shown. Data are presented as mean ± SE. (**D**) A549 cell lysates were immunoprecipitated using anti-C/EBPβ antibodies. Immunocomplexes were analyzed by Western blot with either anti-HDAC1 or -HDAC2 antibodies. IgG was used as a negative control. (**E**) HDAC2-ChIP assay followed by qRT-PCR on putative C/EBPβ binding regions at the *WEE1* promoter was performed. The fold enrichment of HDAC2 occupancy over *GAPDH* exon (negative control) is shown. Data are presented as mean ± SE. (**F**) The C/EBPβ-ChIP or HDAC2-ChIP assay followed by PCR on putative C/EBPβ binding regions at the *WEE1* promoter was performed. The PCR products resolved on 2% agarose gel were visualized. (**G**) A549 cells were co-transfected with a *WEE1* promoter-luciferase construct containing R2, or R3 along with C/EBPβ and/or HDAC2, as indicated, for 48 h, and then luciferase activities were measured. Data are expressed as relative luciferase activity/ug protein standardized by a control pGL3-promoter vector. Data are presented as mean ± SD. Statistical significance was determined using the *t*-test, * *p* < 0.05.

Next, we investigated if either *WEE1* or *CDC25B* was the transcription target of C/EBPβ playing a role in cell cycle regulation. As shown in Figure 5A, in C/EBPβ-knockdown cells, mRNA levels of Wee1 and Cdc25B increased and decreased, respectively, correlated with their protein levels (Figure 5A). With our C/EBPβ-ChIP on chip data (unpublished data) and using a web-based tool for searching transcription factor binding sites, TFSEARCH [54], we predicted four putative C/EBPβ binding regions on the *WEE1* distal promoter, as shown in Figure 5B. We performed a C/EBPβ-ChIP assay and real-time PCR to verify that C/EBPβ bound to −4.7 to −4.9 kB (R3) and −4.4 to −4.5 kB (R2) upstream of the *Wee1* transcription start site (Figure 5C,F). However, we did not find any significant binding of C/EBPβ to the *CD25B* promoter based on our C/EBPβ-ChIP on chip data (unpublished data).

C/EBPβ has been reported to interact with histone deacetylase1 (HDAC1) and repress the activation of the C/EBPα promoter [55]. Our data showed that C/EBPβ interacts with HDAC2, not with HDAC1 in A549 cells (Figure 5D). In addition, the HDAC2-ChIP assay revealed that HDAC2 also bound to the R2 and R3 regions (Figure 5E,F), suggesting that C/EBPβ recruits HDAC2 at the *WEE1* distal promoter. To determine if C/EBPβ in conjunction with HDAC2 repressed *WEE1* promoter activity, we constructed a promoter-reporter vector containing either the R2 or R3 region of the distal *WEE1* promoter. Enforced expression of C/EBPβ or HDAC2 alone reduced *WEE1* promoter-reporter activity and C/EBPβ along with HDAC2 further decreased transcriptional activity of the *WEE1* promoter, indicating that C/EBPβ recruits HDAC2 to the *WEE1* promoter, negatively regulating *WEE1* transcription (Figure 5G). Taken together, we identified Wee1, a mitosis inhibitor, as a novel transcription target gene of C/EBPβ.

### 3.5. C/EBPβ-Knockdown Cells Treated with MK1775 Were Recovered from G_2_/M Delay

**Figure 6:**
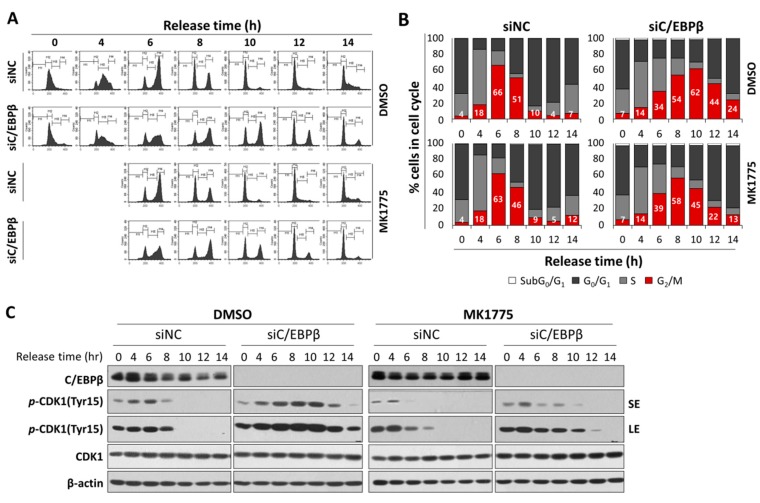
C/EBPβ knockdown cells treated with MK1775 recovered from the delay in the G_2_/M phase. (**A**,**B**) A549 cells transfected with siNC or siC/EBPβ were treated with either DMSO or MK1775 4 h after being released from thymidine double block. Cells were harvested for cell cycle analysis at different time points after release, and DNA contents with PI staining were analyzed using FACS. (**C**) Expression of cell cycle-associated proteins was analyzed in DMSO- or MK1775-treated control cells and C/EBPβ-knockdown cells. Percentage of cells in each cell cycle phase is shown as a bar graph. SE; short exposure, LE; long exposure.

To confirm Wee1 plays an important role in a delay in the G_2_/M cell cycle shown in C/EBPβ deficiency, we examined whether the inhibition of Wee1 could release C/EBPβ-knockdown cells from the delay in the G_2_/M. Four hours after release into the cell cycle at the completion of the thymidine double block, cells were treated with a Wee1 inhibitor, MK1775, or DMSO. MK1775 treatment of control cells had little effect on cell cycle progression (Figure 6A,B). However, Wee1 inhibition of C/EBPβ-knockdown cells diminished accumulation of cell populations in the G_2_/M phase induced by C/EBPβ-deficiency (Figure 6A,B). More specifically, C/EBPβ-knockdown cells accumulated in the G_2_/M phase up to 10 h (62.2%) because of a delay in the G_2_/M phase and decreased to 43. 6% at 12 h after release. However, upon MK-1775 treatment of C/EBPβ-knockdown cells, cell populations in the G_2_/M phase maximally reached up to 57.8% at 8 h, further progressed into the G_0_/G_1_ and S phase, and then decreased to 44.9% and 22% at 10 and 12 h after release, respectively. Consistent with the results in Figure 3 and Figure 4, compared with control cells, Y15-pCDK1 increased in the C/EBPβ-knockdown cells (Figure 6C). Treatment with MK1775 induced a rapid decrease in Y15-pCDK1 with little changes in total levels of CDK1. As MK1775 treatment rescued C/EBPβ-knockdown cells from the delay in the G_2_/M phase, we argue that increased Y15-pCDK1 by Wee1 is responsible for the delay in the G_2_/M phase with defective C/EBPβ function.

### 3.6. C/EBPβ-Knockdown Inhibits Tumor Growth In Vivo

**Figure 7:**
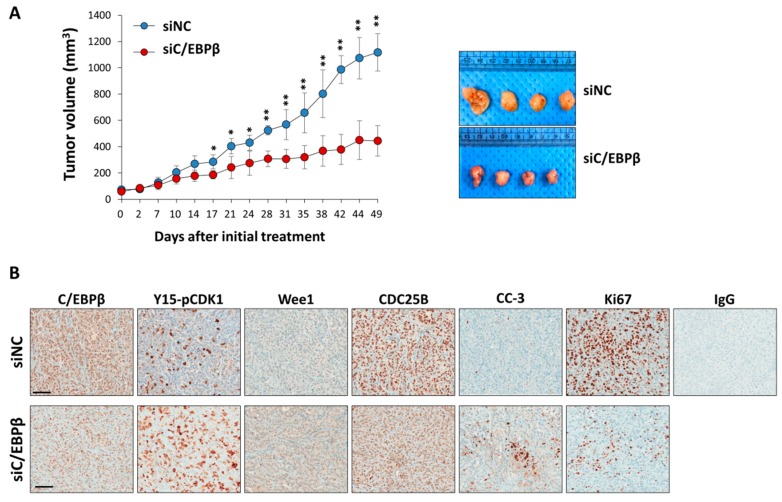
C/EBPβ-knockdown inhibits tumor growth. (**A**) A549 cells (5 × 10^6^) were implanted subcutaneously into athymic nude mice. When tumor size reached 60 to 80 mm^3^, siNC or siC/EBPβ were delivered into the tumors via electroporation once a week for seven weeks. Tumors were measured at the indicated time and tumor volume was calculated as described in Section 2. Photos from siNC- or siC/EBPβ-treated tumors are shown. Similar results were observed in three independent experiments. Data are presented as mean ± SD. The statistical significance was determined using the *t*-test, * *p* < 0.05, ** *p* < 0.01, significantly different from siC/EBPβ-treated tumor volume. (**B**) Immunohistochemical staining for C/EBPβ, Y15-pCDK1, Wee1, Cdc25B, Ki67, and cleaved caspase-3 (CC-3) was conducted with paraformaldehyde-fixed, paraffin-embedded xenograft tumors. Images were captured at a magnification of 400X by using the Aperio ImageScope software. Scale bars: 100 μm.

Since C/EBPβ-knockdown inhibits cell proliferation inducing a delay in the G_2_/M phase of the cell cycle, we tested if C/EBPβ-knockdown reduces the growth of xenograft tumors in vivo. Tumors were produced by injecting A549 cells subcutaneously into the dorsal area of athymic nude mice, and siNC or siC/EBPβ RNA was delivered into the tumors via electroporation. Tumor growth was monitored for seven weeks. As shown in Figure 7A, siC/EBPβ treatment markedly suppressed tumor growth by at least 50% compared with the siNC treatment. Consistent with in vitro results, immunohistochemistry analysis revealed that treatment with siC/EBPβ increased the expression of Y15-pCDK1 and Wee1 while decreasing Cdc25B expression (Figure 7B). C/EBPβ-knockdown tumors displayed lower Ki67 and higher cleaved caspase-3 expression compared with the control. These data indicate that C/EBPβ is important for tumor growth in vivo.

## 4. Discussion

**Figure 8:**
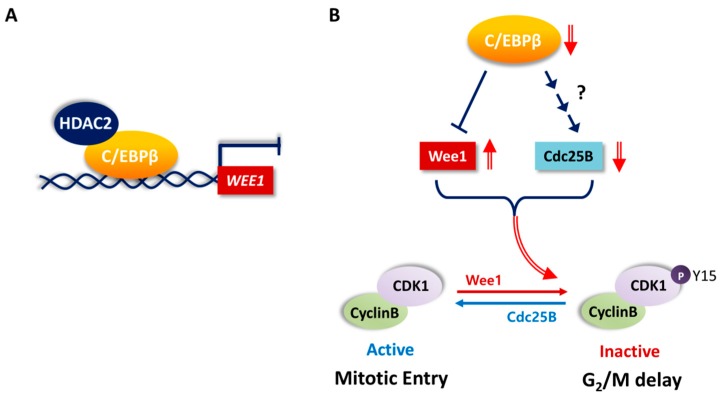
Schematic representation of the potential role of C/EBPβ in the G_2_/M phase of cell cycle progression. (**A**) C/EBPβ represses *WEE1* transcription by directly binding to *WEE1* distal promoter regions and recruiting HDAC2. (**B**) Wee1 and Cdc25B are key regulators of phosphorylation of tyrosine 15 residue of CDK1, which blocks mitotic entry. C/EBPβ activates Cdc25B expression via unknown mechanism but inhibits Wee1 expression. In the absence of C/EBPβ, cells undergo G_2_/M delay displaying increased CDK1 phsophorylation along with increased Wee1 and decreased Cdc25B levels.

Mitosis is regulated by cyclin B/CDK1 [56]. Wee1 kinase phosphorylates CDK1 at Tyr15, and dephosphorylation of this site by Cdc25 is required for the activation of CDK1 and further entry into mitosis. We showed that C/EBPβ is important for the proliferation of NSCLC cells by mediating the G_2_/M transition of cell cycle. C/EBPβ regulated the inhibitory phosphorylation at the Tyr15 residue of CDK1 (Figure 3 and Figure 4). This resulted from the function of C/EBPβ, which increased the expression of Cdc25B phosphatase but inhibited the expression of Wee1 kinase at the mRNA and protein levels (Figure 3, Figure 4 and Figure 5A). The mechanism of regulating Cdc25B expression by C/EBPβ does not seem to be mediated by direct transcriptional activation and is yet to be determined. C/EBPβ bound to two *WEE1* distal promoter regions, −4.7 to −4.9 kB and −4.4 to −4.5 kB, upstream of the transcription start site and repressed transcription through recruiting HDAC2 (Figure 5). These findings are summarized as a schematic diagram in Figure 8. To the best of our knowledge, this is the first report that Wee1, a key regulator of G_2_/M progression, is a transcriptional target of C/EBPβ. Finally, we showed that a Wee1 inhibitor, MK-1775, significantly recovered C/EBPβ-knockdown cells from the delay in the G_2_/M phase of the cell cycle (Figure 6). Taken together, these results indicate that C/EBPβ is a transcriptional regulator of Wee1, mitosis inhibitor protein, ultimately regulating the G_2/_M phase of the cell cycle progression.

Cell cycle proteins need to be tightly controlled temporally and spatially. Wee1 increases during the S and G_2_ phase to block premature mitotic entry and then decreases during the M phase of the cell cycle [57]. Regulation of the Wee1 protein has been extensively studied and several mechanisms of modulating its kinase activity and protein levels have been demonstrated [58]. For example, Wee1 kinase activity is regulated by Akt-mediated phosphorylation on Ser642 and phosphorylated Wee1 binds to 14–3-3θ and translocates to the cytoplasm, resulting in G_2_/M cell cycle progression [59]. In late G_2_ phase, phosphorylation of Wee1 by CDK1 and polo-like kinase 1 creates a phosphodegron which targets Wee1 for SCF–β-TrCP ubiquitin ligase-mediated proteasomal degradation [60]. However, transcriptional regulation of *WEE1* is not well known. *WEE1* transcription was shown to be repressed by direct binding of kruppel-like factor 2 (KLF2) and chromodomain helicase DNA binding protein 5 (CHD5) and activated by c-Fos/activator protein-1 (AP-1) [61,62,63]. Our results also demonstrated that Wee1 is downregulated by C/EBPβ via direct binding to the distal promoter and it is required for the G_2_/M cell cycle progression in lung cancer cells.

C/EBPβ positively regulated the proliferation of various NSCLC cells regardless of NSCLC subtypes or gene mutation status of *EGFR* or *K-RAS*, whereas it did not have a substantial effect on immortalized lung epithelial cells (Figure 2). Even if we identified Wee1 and Cdc25B as downstream players of C/EBPβ in the cell cycle regulatory role, the upstream cue, possibly altered in NSCLC, remains to be determined.

In our study, control cells after release from the thymidine double block progressed to the S phase rapidly reaching the peak levels in 2 h, and majority of them moved into the G_2_/M phase in another 4 h (Figure 4A,B and Figure 6A,B). However, in C/EBPβ-knockdown cells, G_1_/S transition was delayed and significant population of cells remained in the G_0_/G_1_ phase (Figure 4A,B), suggesting that G_1_/S cell cycle arrest is also induced in the absence of C/EBPβ. Consistent with our observation, C/EBPβ-deficient human endometrial stromal cells underwent G_1_/S arrest shown by the reduction of BrdU incorporation after release from thymidine double block. In those cells, cyclin E was downregulated and thereby cyclin E-CDK2 was not functional for G_1_/S phase progression [64]. C/EBPβ has been shown to cooperate with E2F in activating transcription of E2F target genes involved in the G_1_/S transition by recruiting CBP/p300 coactivator [65].

C/EBPs are expressed in human lung epithelium and they play roles in lung development and differentiation [66]. C/EBPα-deficient mice displayed hyper-proliferative alveolar type II cells and a defect in alveolar architecture and suffered from respiratory distress [67,68], whereas lung phenotypes of C/EBPβ knockout mice were not specified [66]. Even if C/EBPβ is involved in the lung-specific gene expression in lung epithelial cells [69,70], it does not seem to be essential in basal lung development or differentiation, possibly due to functional redundancy with other C/EBP family members, as described in liver and mouse skin [71,72]. Rather acute lung injury is induced by lipopolysaccharide (LPS)-induced C/EBPβ mRNA [73], suggesting C/EBPβ might play a distinct role in pathological status.

Loss in the function mutations were found in acute myeloid leukemia, suggesting C/EBPα as potential human tumor suppressor [74]. C/EBPα has been reported to be down-regulated in more than 40% of human primary lung cancers [70,75], and it seems to be associated with increased DNA methylation of *C/EBPA* promoter [76]. More recently, the oncogenic role of C/EBPβ has been suggested in human cancers, but how it contributes to tumorigenesis or tumor progression needs to be determined. We found that C/EBPβ protein is up-regulated in NSCLCs (Figure 1). Additionally, the delivery of siRNA against C/EBPβ into xenografted mouse tumors effectively inhibited tumor growth (Figure 7). Considering the notion that the function of C/EBPβ is not vital in the lung, C/EBPβ could be an attractive target for cancer therapy alone or in combination.

## Figures and Tables

**Table 1:** Immunohistochemistry (IHC) scoring of C/EBPβ.

Score	Normal *n* (%)	Tumor *n* (%)	Metastasis *n* (%)
0	47 (69.1)	31 (32.6)	1 (11.1)
1	20 (29.4)	36 (37.9)	3 (33.3)
2	1 (1.5)	10 (10.5)	2 (22.2)
3	0 (0)	13 (13.7)	3 (33.3)
4	0 (0)	5 (5.3)	0 (0)
Total	68 (100)	95 (100)	9 (100)

**Table 2:** Summary of C/EBPβ expression and clinicopathological feature in the lung cancer patients.

	C/EBPβ Expression				
	Negative *n* (%)	Positive *n* (%)	Total *n* (%)	*p* Value	
**Sex**					
Male	23 (31)	52 (69)	75 (100)	0.4343	
Female	8 (40)	12 (60)	20 (100)		
**Age**					
≤57	11 (31)	24 (69)	35 (100)	0.8505	
>57	20 (33)	40 (67)	60 (100)		
**Clinical Stage**					
I	10 (27)	27 (73)	37 (100)	0.2305	
II	12 (32)	25 (68)	37 (100)		
III	9 (43)	12 (57)	21 (100)		
**N classification**					
0	15 (28)	38 (72)	53 (100)	0.2204	
1	8 (33)	16 (67)	24 (100)		
2	8 (44)	10 (66)	18 (100)		
**T classification**					
1	1 (13)	7 (87)	8 (100)	0.3039	
2	23 (33)	47 (67)	70 (100)		
3	5 (46)	6 (54)	11 (100)		
4	2 (33)	4 (67)	17 (100)		
**Histology**					
Adenocarcinoma	13 (46)	15 (54)	28 (100)	0.0180 *	0.0256 ^†^
Squamous carcinoma	15 (31)	34 (69)	49 (100)		0.2941 ^#^
Other NSCLC	3 (17)	15 (83)	18 (100)		

* Adenocarcinoma vs. squamous cell carcinoma, ^†^ Adenocarcinoma vs. other NSCLC, ^#^ Squamous cell carcinoma vs. other NSCLC, * ^†^ significantly different between two groups, *p* < 0.05. C/EBPβ staining was analyzed with 95 primary NSCLC tissues from 95 NSCLC patients.

## Supplementary Materials

The following are available online at https://www.mdpi.com/2073-4409/8/2/145/s1. Figure S1: Expression and gene alteration of C/EBPβ in human lung cancers. Figure S2: Overall survival (OS) and post-progression survival (PPS) of lung cancer patients. Table S1: Histological subtypes and gene mutation status of NSCLC cell lines. Figure S3: FACS analysis of mitotic cells using mitotic marker, phospho-Histone H3 (Ser10).
