# Supplementary material for: C/EBPβ Is a Transcriptional Regulator of Wee1 at the G2/M Phase of the Cell Cycle
Source: Cells. 2019 Feb 11;8(2):145. doi: 10.3390/cells8020145 (PMC6407104; doi:10.3390/cells8020145)
Supplement: Supplementary file 1 [file cells-08-00145-s001.pdf]

cells

MDPI

Supplementary Materials

Methods

FACS Analysis of Mitotic Cells

To detect mitotic cells, cells were fixed in ice-cold methanol for 16 h, then incubated with phospho-Histone H3 (Ser10) (1:500, ab14955, Abcam, Cambridge, UK) for 1 h at room temperature (RT). After washing with 1% bovine serum albumin in phosphate-buffered saline, cells were stained with fluorescein-5-isothiocyanate (FITC)-conjugated secondary antibody for 30 min at RT and DNA was stained with propidium iodide (PI). Finally, samples were analyzed by flow cytometry (FACSCaliber; Becton Dickinson, Franklin Lakes, NJ, USA).

Figure S1. Expression and gene alteration of C/EBPβ in human lung cancers

A

Diagnosis	C/EBPβ score					
	0	1	2	3	4	total
Squamous cell carcinoma	15	19	4	8	3	49
Adenocarcinoma	13	12	2	1	-	28
Large cell carcinoma	2	1	2	3	-	8
Adenosquamous carcinoma	1		1	1		3
Carcinosarcoma			1		1	2
Mucoepidermoid carcinoma		4				4
Adenoid cystic carcinoma				1		1

B

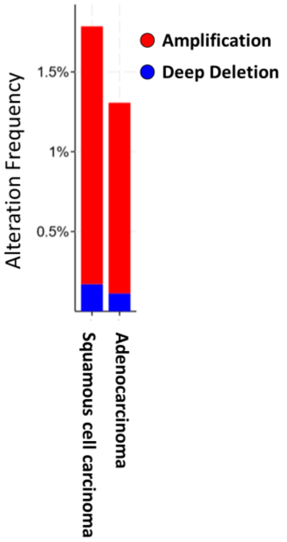

(A) Detailed score of C/EBPβ expression in NSCLC patient tissues. (B) The alterations of C/EBPβ gene were analyzed using cBioPortal Cancer Genomics [1, 2]. C/EBPβ gene is altered in 1.74% (amplification 1.57% and deep deletion 0.17%) of squamous cell carcinoma and in 1.32% (amplification 1.22% and deep deletion 0.1%) of adenocarcinoma.

Figure S2. Overall survival (OS) and post-progression survival (PPS) of lung cancer patients

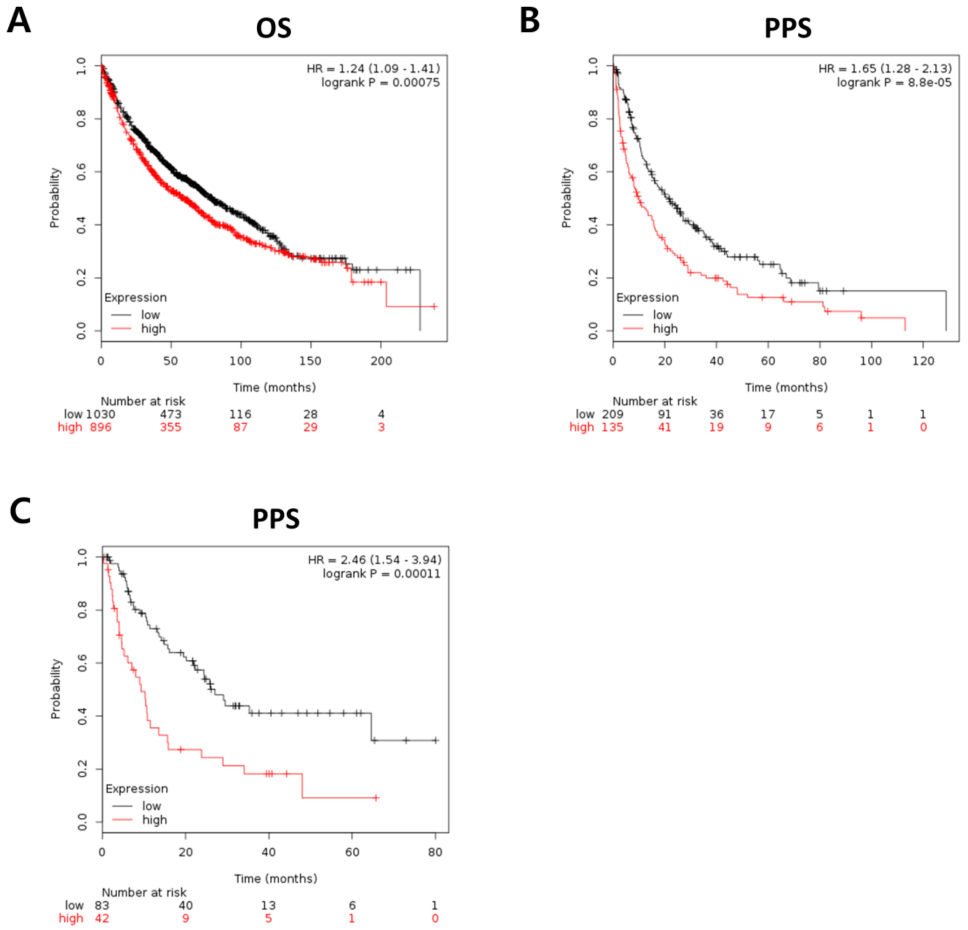

The association between C/EBPβ mRNA expression and (A) OS, (B) PPS of total lung cancer patients or (C) PPS of adenocarcinoma patients was analyzed using the Kaplan-Meier Plotter [3]. Kaplan-Meier analysis was performed in whole dataset.

Table S1. Histological Subtypes and gene mutation status of NSCLC cell lines.

Cell line	Histological type	EGFR mutation	KRAS mutation	Reference
A549	Adenocarcinoma (AC)	WT	G12S	[4]
Calu-6	Anaplastic carcinoma	WT	Q61H	[5]
H1299	Large cell carcinoma	WT	WT/NRAS Q61K	[4]
H1703	Adenocarcinoma	WT	WT	[4]
H1975	Adenocarcinoma	L858R, T790M	WT	[5]
H23	Adenocarcinoma	WT	G12C	[4]
H460	Large cell carcinoma	WT	Q61H	[4]
HCC2279	Adeno-squamous cell carcinoma	delE746-A750	WT	[6]
H522	Adenocarcinoma	WT	WT	[4]
A427	Adenocarcinoma	WT	G12D	[4]
Calu-3	Adenocarcinoma	WT	WT	[5]
H358	Bronchioalveolar Carcinoma (AC)	WT	G12C	[4]
HCC827	Adenocarcinoma	delE746-A750	WT	[7]
HCC95	Squamous cell carcinoma	WT	WT	[4]
HCC1588	Squamous cell carcinoma	WT	WT	[6, 8]

Figure S3. FACS analysis of mitotic cells using mitotic marker, phospho-Histone H3 (Ser 10).

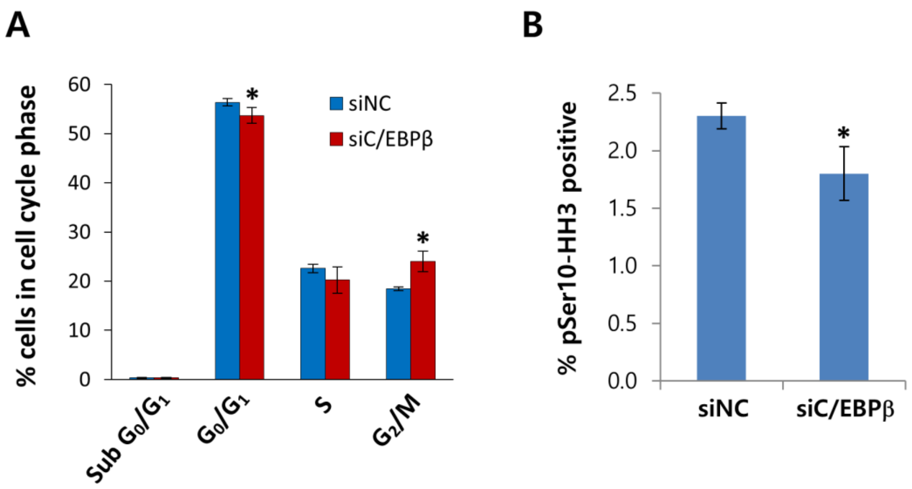

Cells were fixed 48 h after siRNA transfection and stained with anti-phospho-histone H3 (Ser10) to detect mitotic cells and PI for DNA content. A. Populations of each cell cycle of control and C/EBPβ-knockdown cells are shown. B. Mitotic populations of control and C/EBPβ-knockdown cells are shown. Data are presented as mean ± SD. Statistical significance was determined using the *t*-test, * $p < 0.05$.

References

1. Cerami, E.; Gao, J.; Dogrusoz, U.; Gross, B. E.; Sumer, S. O.; Aksoy, B. A.; Jacobsen, A.; Byrne, C. J.; Heuer, M. L.; Larsson, E.; Antipin, Y.; Reva, B.; Goldberg, A. P.; Sander, C.; Schultz, N., The cBio cancer genomics portal: an open platform for exploring multidimensional cancer genomics data. *Cancer Discov* **2012,** *2* (5), 401-4.
2. Gao, J.; Aksoy, B. A.; Dogrusoz, U.; Dresdner, G.; Gross, B.; Sumer, S. O.; Sun, Y.; Jacobsen, A.; Sinha, R.; Larsson, E.; Cerami, E.; Sander, C.; Schultz, N., Integrative analysis of complex cancer genomics and clinical profiles using the cBioPortal. *Sci Signal* **2013,** *6* (269), pl1.
3. Gyorffy, B.; Surowiak, P.; Budczies, J.; Lanczky, A., Online survival analysis software to assess the prognostic value of biomarkers using transcriptomic data in non-small-cell lung cancer. *PloS one* **2013,** *8* (12), e82241.
4. Blanco, R.; Iwakawa, R.; Tang, M.; Kohno, T.; Angulo, B.; Pio, R.; Montuenga, L. M.; Minna, J. D.; Yokota, J.; Sanchez-Cespedes, M., A gene-alteration profile of human lung cancer cell lines. *Human mutation* **2009,** *30* (8), 1199-206.
5. Forbes, S.; Clements, J.; Dawson, E.; Bamford, S.; Webb, T.; Dogan, A.; Flanagan, A.; Teague, J.; Wooster, R.; Futreal, P. A.; Stratton, M. R., COSMIC 2005. *British journal of cancer* **2006,** *94* (2), 318-22.
6. Wistuba, II; Bryant, D.; Behrens, C.; Milchgrub, S.; Virmani, A. K.; Ashfaq, R.; Minna, J. D.; Gazdar, A. F., Comparison of features of human lung cancer cell lines and their corresponding tumors. *Clinical cancer research : an official journal of the American Association for Cancer Research* **1999,** *5* (5), 991-1000.
7. Alvarez, J. V.; Greulich, H.; Sellers, W. R.; Meyerson, M.; Frank, D. A., Signal transducer and activator of transcription 3 is required for the oncogenic effects of non-small-cell lung cancer-associated mutations of the epidermal growth factor receptor. *Cancer research* **2006,** *66* (6), 3162-8.
8. Yamamoto, H.; Shigematsu, H.; Nomura, M.; Lockwood, W. W.; Sato, M.; Okumura, N.; Soh, J.; Suzuki, M.; Wistuba, II; Fong, K. M.; Lee, H.; Toyooka, S.; Date, H.; Lam, W. L.; Minna, J. D.; Gazdar, A. F., PIK3CA mutations and copy number gains in human lung cancers. *Cancer research* **2008,** *68* (17), 6913-21.
